# Test–retest reliability of tip, key, and palmar pinch force sense in healthy adults

**DOI:** 10.1186/s12891-020-3187-7

**Published:** 2020-03-26

**Authors:** Lin Li, Yanxia Li, Changhong Wu, Xinyan Zhang

**Affiliations:** 1 Department of Physical Education, Renmin University of China, Beijing, People’s Republic of China; 2 College of Physical Education, Langfang Teachers University, Langfang, Hebei 065000 People’s Republic of China; 3 School of Sociology and Population Studies, Renmin University of China, Beijing, People’s Republic of China

**Keywords:** Proprioception, Force sense, Tip pinch, Key pinch, Palmar pinch, Test–retest reliability

## Abstract

**Background:**

No previous studies have investigated the test–retest reliability of tip, key, and palmar pinch force sense in healthy adults. The present study explores the test-retest reliability of tip, key, and palmar pinch force sense for different force levels in healthy adults during an ipsilateral force reproduction task.

**Methods:**

Fifty-six healthy subjects were instructed to produce varying levels of reference forces (10, 30, and 50% maximal voluntary isometric contraction (MVIC)) using three types of pinches (tip pinch, palmar pinch, and key pinch) and to reproduce these forces using the same hand. The subjects were tested twice by the same experienced testers, 1 week apart.

**Results:**

Based on the high values of the intraclass correlation coefficient (ICC), the tip pinch (0.783–0.895) and palmar pinch (0.752–0.903) force sense tests demonstrated good reliability for all the variables. The ICCs for the key pinch (0.712–0.881) indicated fair to good relative test-retest reliability.

**Conclusion:**

1) This study demonstrates that high test-retest reliability of tip, key, and palmar pinch force sense in healthy adults can be achieved using standardized positioning and the proposed approach. 2) According to the reliability measurements, 30 and 50% maximal voluntary isometric contraction (MVIC) are the most reliable pinch force sense levels.

## Background

Proprioception is critical for accurate movement. It enables communication from the periphery to the central nervous system (CNS), which is required for the body to acquire joint position awareness and maintain a desired postural orientation and overall position in space. There are three types of conscious proprioceptive senses: kinesthesia, joint position sense, and force sense [1, 2]. All of them, especially force sense, play a role in good neuromuscular control. Force sense is defined as the ability to detect and interpret forces applied to or generated within a joint [3]. Force sense is measured by the performance accuracy of individuals during force reproduction tasks, which are defined as tasks in which individuals are instructed to produce target forces and reproduce these forces [4].

Different types of pinch grips (tip pinch, palmar pinch, key pinch [5–9]) or combinations of these pinch grips with different force levels are frequently used in workplaces. Workers in various occupations, such as mechanics, repair persons, and engineers, must maintain various pinch grips at constant, submaximum force levels using various hand tools and equipment when performing a wide range of operations, from the assembly of small electronic parts to the assembly of large airplanes. Spontaneously pinching an object is a complex motor task since a sufficient pinch force must be applied to prevent slipping and, at the same time, excessive force must be avoided to prevent the object from being crushed or the person from experiencing unnecessary fatigue. Repeated and unnecessarily high pinch forces have been previously identified as risk factors for the development of musculoskeletal disorders (MSDs), including carpal tunnel syndrome (CTS) [10, 11], tendonitis [12], and epicondylitis [13].

Test-retest reliability is clinically important for the accurate interpretation of follow-up results. If a measurement procedure or tool has good test-retest reliability, accurate comparisons can be made over time intervals. Reliable test results allow clinicians to draw conclusions that are minimally affected by external factors, thereby reducing the chances of error. Previous studies have investigated the test-retest reliability of the force sense test in ankles [14], knees [15], hips [16], shoulders [17], and hand grips [18]. To the best of our knowledge, no previous studies have investigated the reliability of the pinch force sense test. Compared with other segments in the human body, the finger has few muscles and is easily fatigued. In addition, the sensing and control of forces are complicated by the coordination of the two or three fingers (thumb, index, and long finger) that are involved in the pinch grip. Therefore, the objective of this study was to investigate the test-retest reliability of the pinch force sense test in healthy adults.

## Methods

### Participants

Fifty-six healthy subjects (31 women and 25 men, age 21.7 ± 5.9 years, weight 62.6 ± 12.7 kg, height 168.4 ± 8.1 cm, all right-handed) were tested. The sample size was based on the recommendations of Fleiss, who indicated that 15 to 20 subjects are sufficient for estimating the reliability of a quantitative variable [19]. Hand dominance was indicated by the subjects based on the hand used for writing. The exclusion criteria included 1) a long-standing history of highly skilled motor activity, such as playing a musical instrument or basketball [20], 2) a prior hand surgery, and 3) the presence of hand pain or a hand pathology. The objectives of the study and the experimental procedure were carefully explained to the subjects, and written consent forms were obtained [21]. Authorization to carry out this research was granted by our university’s ethics review board.

### Apparatus

Strength tests and force reproduction estimations were conducted using an electronic digital force dynamometer (pinch analyzer; Kjyl Technologies, CHN). Calibration of the instrument was performed by the manufacturer. Preliminary testing was also performed to prevent errors during the study. The pinch span of the dynamometer was adjustable and was set at 2 cm in the strength tests and force reproduction estimations. For the present study, the sampling frequency was fixed at 100 Hz. Based on the pinch analyzer, a protocol in which the pinch force sense is measured was developed.

### Protocol

**Fig. 1:**
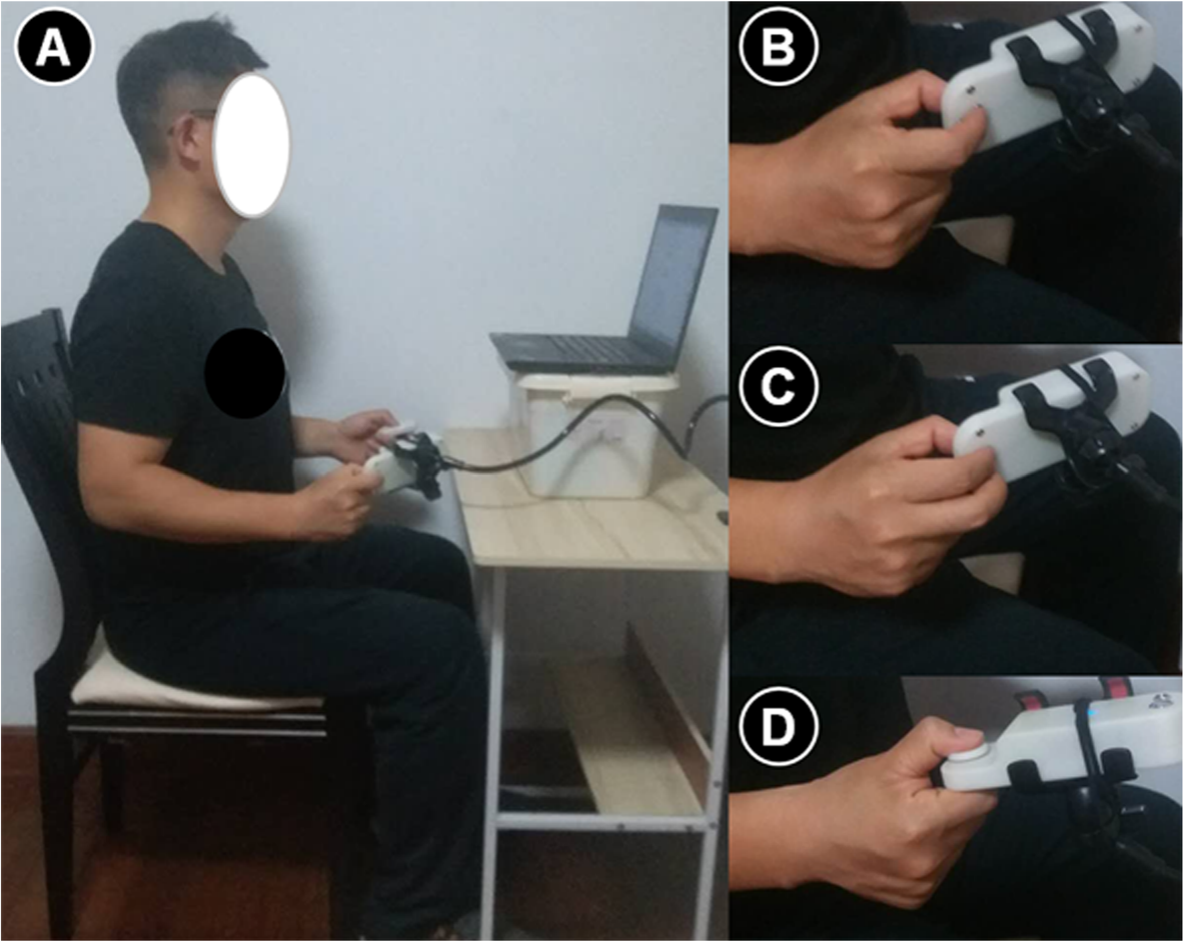
The standardized positioning (**a**) used for tip (**b**), palmar (**c**), and key pinch (**d**) force sense measurement

The study was performed in a quiet room to ensure that auditory distractions were properly minimized [22]. The subjects sat in a chair that was located approximately 60 cm from a 14-in. LCD monitor, and they assumed a whole-body posture that was in line with the American Society of Hand Therapists guidelines: the upper arm was positioned vertically, the elbow was flexed at 90°, and the forearm and wrist were set in neutral positions [23] (Fig. 1a). The subjects performed isometric pinching tasks with three types of pinches: the tip pinch, palmar pinch, and key pinch. For the tip pinch, the tip of the thumb touched the index fingertip while the other fingers are fully flexed (Fig. 1b). For the palmar pinch, the pad of the thumb touched the pads of the index and long fingers (Fig. 1c). For the key pinch, the pad of the thumb touched the lateral aspect of middle phalanx of the index finger (Fig. 1d) [5, 24]. The participants were asked to maintain this same arm configuration throughout the testing period. They were able to view their pinch force output on a 14-in. monitor. A PC desktop equipped with a customized MVIC testing program and force reproduction task program (Kjyl Technologies, CHN) was used for data acquisition and processing.

#### MVIC test

The subjects were instructed to use three different types of pinches (tip, palmar, and key pinch). The types of pinches were presented in a random order. They performed warm-up activities for the type of pinch force that was tested first. The warm-up activities consisted of three repetitions of achieving a submaximal pinch grip force, as measured by the dynamometer [25]. The subjects were instructed to apply a maximal pinch force on the dynamometer after the warm-up activities. This test was repeated twice for each type of pinch grip, and the highest value was recorded as the pinch strength [26]. To minimize the effect of fatigue, three-minute resting periods were allowed between tests.

#### Force reproduction task

**Fig. 2:**
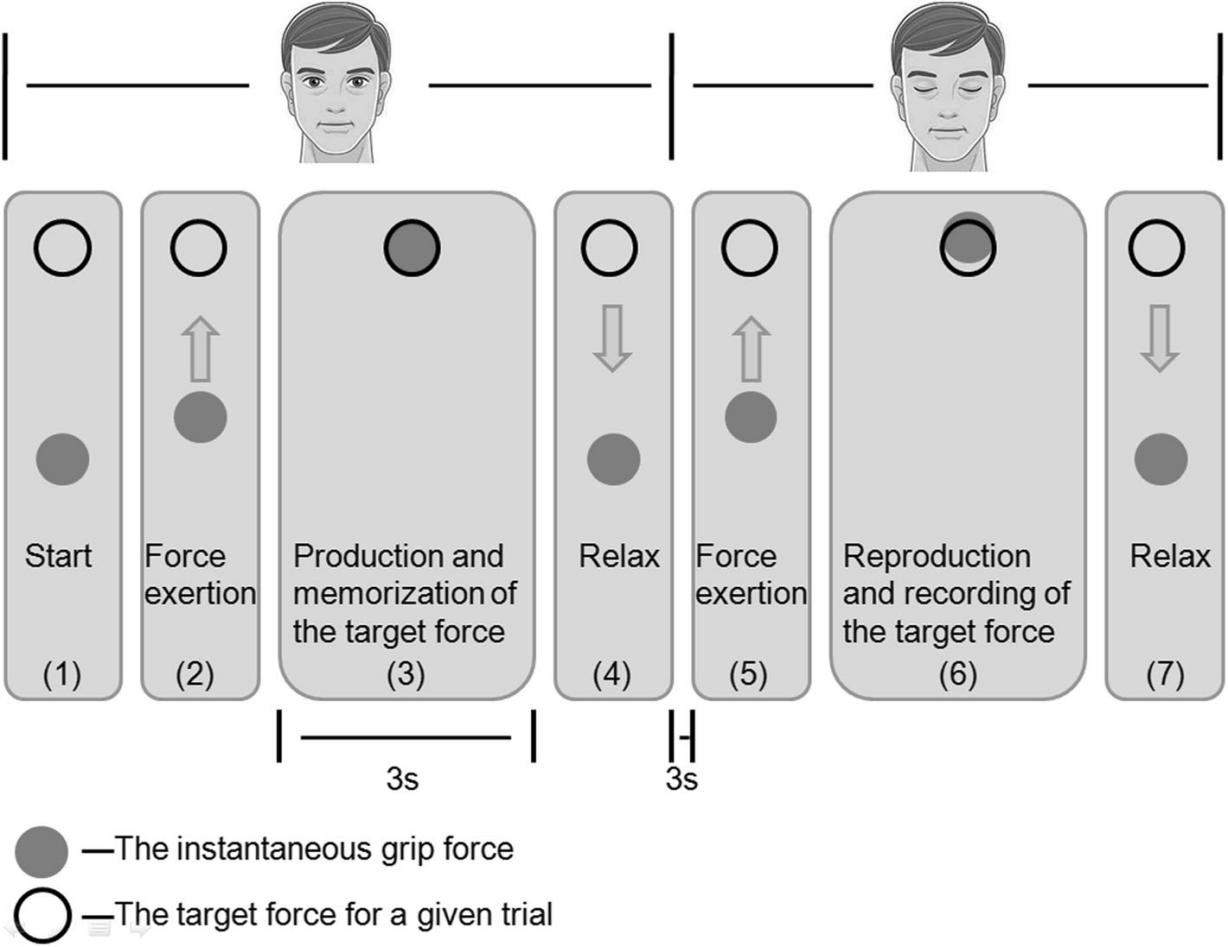
Schematic of the computer output displayed on the monitor to guide the subject to the target force

Force sense was measured by the accuracy of the subjects in the reproduction of force tasks. The subjects were informed on how to perform the test as they watched a visual demonstration on a computer monitor. A screen with a black circle was shown to the subjects using proprietary C++ software. The black circle signified the target force for a given trial. A gray dot then appeared on the screen, indicating the instantaneous pinch force (Fig. 2). The subjects were required to apply a target force *T* with a pinch grip for 3 s, and they were asked to remember the applied force. They were then given a verbal cue to relax and close their eyes. After a duration of 3 s, the subjects were required to replicate the previous force using the same fingers without any visual feedback. When they believed that the force level was equivalent to the previous one, the subjects pressed a trigger using the other hand, and the computer recorded the force exerted (*R*). The subject was again requested to relax. The subjects performed a standardized warm-up that consisted of three repetitions of the test procedure for familiarization with the apparatus and estimation process and to promote relaxation. Three different types of pinches (tip, palmar, and key pinch) and forces (10, 30, and 50% MVIC) were replicated by the subject, and three contractions were reproduced at each force level. The target forces were presented in a random order. To avoid fatigue, there was a 30-s rest period at the end of each trial, and the subjects were allowed to rest for 2 to 3 min after each set of 5 trials to promote attentiveness during the tasks [27]. Each subject performed 9 trials on two occasions (session 1 and session 2), which took place at approximately the same time of day 1 week apart, with the same experienced tester in the same laboratory to evaluate the reliability and measurement precision of the pinch force sense [27, 28]. The trials were repeated in a randomized order that differed from that used for the first sequence of 9 trials. The participants self-reported that they did not develop any health-related/clinical/functional modifications between the test and the retest. In addition, the participants were instructed to avoid participation in physical activity immediately before the test session and between the test and the retest to prevent fatigue from influencing the testing.

### Data analysis

There were two dependent variables for the force sense errors: absolute error (AE) [29–35] and constant error (CE) [36–40]. AE is an assessment of the overall error, and CE reflects the directionality of the errors (overshoot or undershoot). These parameters were calculated using the following equations:

1 $$ AE=\frac{\sum_{i=1}^3\left|{R}_i-T\right|}{3\bullet T}\times 100\%,\left(i=1,2,3\right), $$

2 $$ CE=\frac{\sum_{i=1}^3\left({R}_i-T\right)}{3\bullet T}\times 100\%,\left(i=1,2,3\right), $$

where *R*_*i*_ is the reproduction force for the *i*th trial and *T* is the target position.

Prior to the analysis, the data were tested for normal distributions using the Kolmogorov–Smirnov test. In the literature, there are contradictory opinions on the best method to calculate reliability or measurement error [41], and it has been stated that a single analysis is inadequate [42, 43]. Therefore, numerous statistical methods were used to determine test-retest reliability: 1) mean difference with a 95% confidence interval (CI) [28] and 2) intraclass correlation coefficient (ICC) [44] with 95% CI [45] estimates were performed based on 2-way mixed effects, absolute-agreement, and single-measurement models [46]. The criteria used to assess and accept the ICC values were as follows: poor - 0.00 to 0.39; fair - 0.40 to 0.74; and good - 0.75 to 1.00 [47]. To assess the systematic differences between the two sessions, a paired *t*-test was performed [48]. 3) The standard error of the measurement (SEM) formula was used to determine the absolute index of reliability [43]. SEM is expressed in actual units and is not influenced by between-subject variability. A high SEM indicates a high level of error, which is indicative of the nonreproducibility of the tested values. 4) The Bland and Altman method of assessing agreement for individual subjects was used; a scatterplot of the differences between session 1 and session 2 (session 2 – session 1) plotted against their mean with 95% limits of agreement (LOA) (LOA = mean difference ± 1.96 SD) was created [49]. If the 95% confidence interval (CI) included the value “0”, then there was no significant variation in the mean [28, 50]. Statistical analyses were performed using SPSS 22.0 (SPSS Inc.). All data are represented as the mean ± SD, and *P* < 0.05 was considered to be statistically significant.

## Results

Tables 1, 2 and 3 show the mean AE and CE and mean difference with 95% CI in relation to the reliability statistics, including the ICC with 95% CI, SEM, and 95% LOA between the test and retest for the three types of pinches at three force levels. The ICCs of AE and CE for the tip pinch (0.783 to 0.895) and palmar pinch (0.752 to 0.903) indicate good relative test-retest reliability. The ICCs of AE and CE for the key pinch ranged from 0.712 to 0.881, indicating fair to good relative test-retest reliability. The SEMs at 30 and 50% MVIC were lower than that at 10% MVIC.

**Table 1:** Test-retest reliability results of the tip pinch force sense

	Force level	Session 1 (%)	Session 2 (%)	Mean difference (%)	ICC (95% CI)	SEM (%)	95% LOA (%)
AE	10% MVIC	47.4 ± 33.4	44.3 ± 27.8	3.1 ± 19.9	0.88 (0.80–0.93)	6.86	−36.0 - 42.2
	30% MVIC	13.7 ± 8.3	14.5 ± 8.1	−0.8 ± 6.9	0.78 (0.63–0.87)	3.23	−14.4 - 12.7
	50% MVIC	14.3 ± 8.1	13.1 ± 7.6	1.2 ± 6.6	0.78 (0.63–0.87)	3.07	− 11.8 - 14.2
CE	10% MVIC	43.6 ± 37.4	41.3 ± 31.3	2.4 ± 21.4	0.90 (0.82–0.94)	6.94	−39.5 - 44.3
	30% MVIC	4.5 ± 14.6	4.6 ± 14.7	0.0 ± 9.6	0.88 (0.80–0.93)	3.33	−18.9 - 18.8
	50% MVIC	−7.3 ± 13.3	−7.5 ± 11.3	0.2 ± 8.6	0.86 (0.77–0.92)	3.19	−16.7 - 17.1

Mean absolute error (AE), constant error (CE), mean difference, intraclass correlation coefficient (ICC) with 95% confidence interval (CI), standard error of the measurement (SEM), and 95% limits of agreement (LOA) between the test and retest of the tip pinch at three force levels

**Table 2:** Test-retest reliability results of the key pinch force sense

	Force level	Session 1 (%)	Session 2 (%)	Mean difference (%)	ICC (95% CI)	SEM (%)	95% LOA (%)
AE	10% MVIC	37.4 ± 29.9	42.1 ± 32.3	−4.6 ± 20.5	0.88 (0.79–0.93)	7.24	−44.8 - 35.5
	30% MVIC	14.9 ± 8.3	13.7 ± 7.4	1.1 ± 7.4	0.71 (0.51–0.83)	3.99	−13.4 - 15.7
	50% MVIC	14.7 ± 9.9	14.1 ± 7.9	0.5 ± 6.3	0.86 (0.76–0.92)	2.33	− 11.7 - 12.8
CE	10% MVIC	34.0 ± 32.8	38.2 ± 35.7	−4.1 ± 22.2	0.88 (0.8–0.93)	7.68	−47.7 - 39.5
	30% MVIC	1.9 ± 16.1	1.9 ± 14.3	0.1 ± 10.6	0.86 (0.77–0.92)	3.90	−20.7 - 20.8
	50% MVIC	− 11.9 ± 12.4	− 10.8 ± 11.1	− 1.1 ± 7.8	0.88 (0.79–0.93)	2.72	−16.3 - 14.1

Mean absolute error (AE), constant error (CE), mean difference, intraclass correlation coefficient (ICC) with 95% confidence interval (CI), standard error of the measurement (SEM), and 95% limits of agreement (LOA) between the test and retest of the key pinch at three force levels

**Table 3:** Test-retest reliability results of the palmar pinch force sense

	Force level	Session 1 (%)	Session 2 (%)	Mean difference (%)	ICC (95% CI)	SEM (%)	95% LOA (%)
AE	10% MVIC	39.8 ± 34.5	43.5 ± 32.4	− 3.7 ± 26.8	0.81 (0.68–0.89)	11.69	−56.2 - 48.9
	30% MVIC	12.4 ± 7.7	13.8 ± 10.4	− 1.4 ± 8.2	0.75 (0.58–0.85)	4.06	− 17.4 - 14.6
	50% MVIC	16.0 ± 9.1	14.3 ± 9.2	1.6 ± 6.5	0.85 (0.74–0.91)	2.55	−11.2 - 14.5
CE	10% MVIC	35.0 ± 39.1	37.7 ± 38.2	−2.7 ± 28.5	0.84 (0.73–0.91)	11.29	−58.6 - 53.3
	30% MVIC	2.4 ± 12.8	0.0 ± 15.9	2.4 ± 10.3	0.85 (0.74–0.91)	4.01	−17.8 - 22.7
	50% MVIC	−10.6 ± 13.8	−10.4 ± 12.3	− 0.2 ± 7.8	0.90 (0.83–0.94)	2.43	−15.5 - 15.2

Mean absolute error (AE), constant error (CE), mean difference, intraclass correlation coefficient (ICC) with 95% confidence interval (CI), standard error of the measurement (SEM), and 95% limits of agreement (LOA) between the test and retest of the palmar pinch at three force levels

**Fig. 3:**
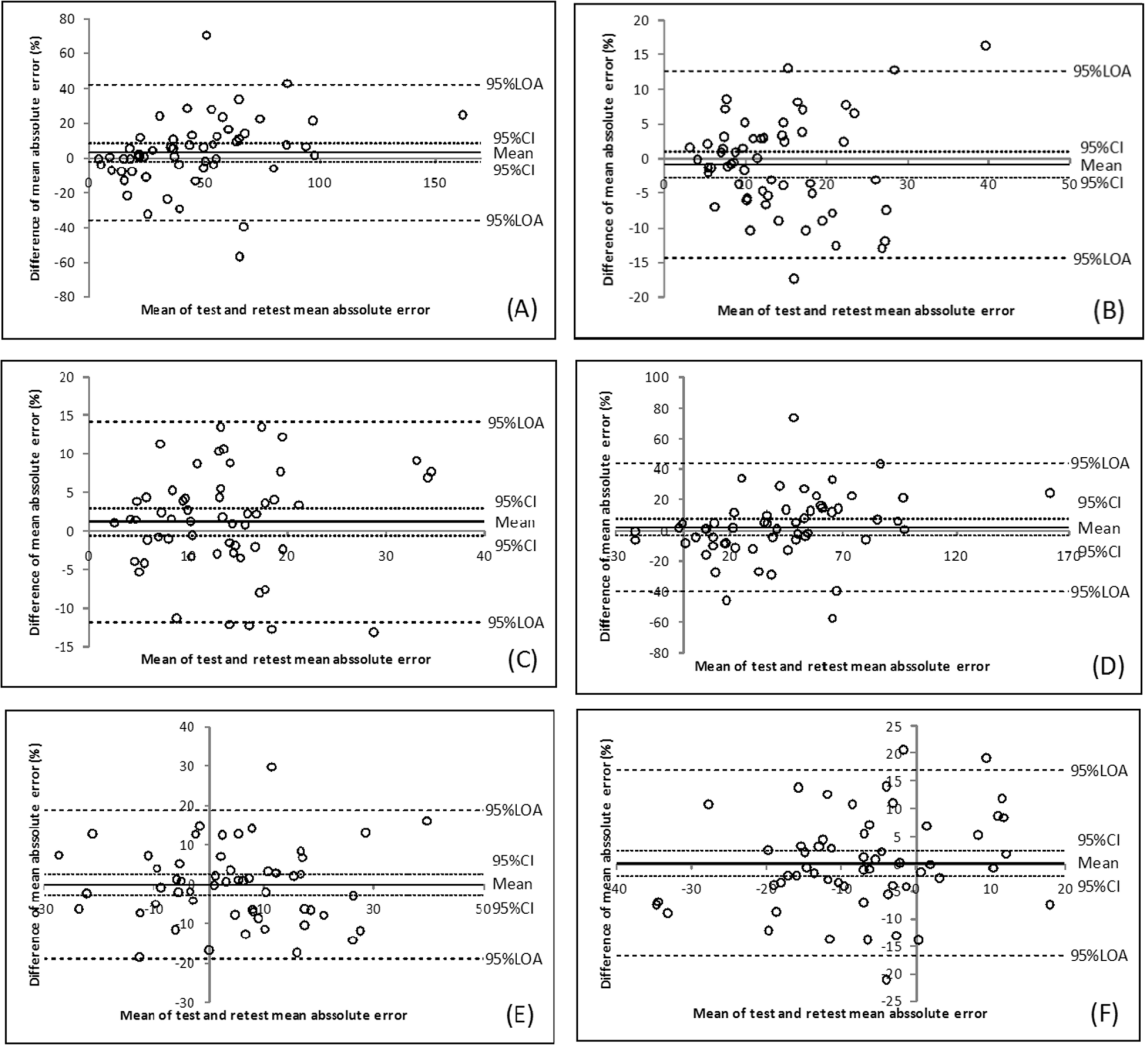
Bland–Altman plots for the AE at 10% MVIC (**a**), 30% MVIC (**b**), and 50% MVIC (**c**) and for the CE at 10% MVIC (**d**), 30% MVIC (**e**), and 50% MVIC (**f**) of the tip pinch

**Fig. 4:**
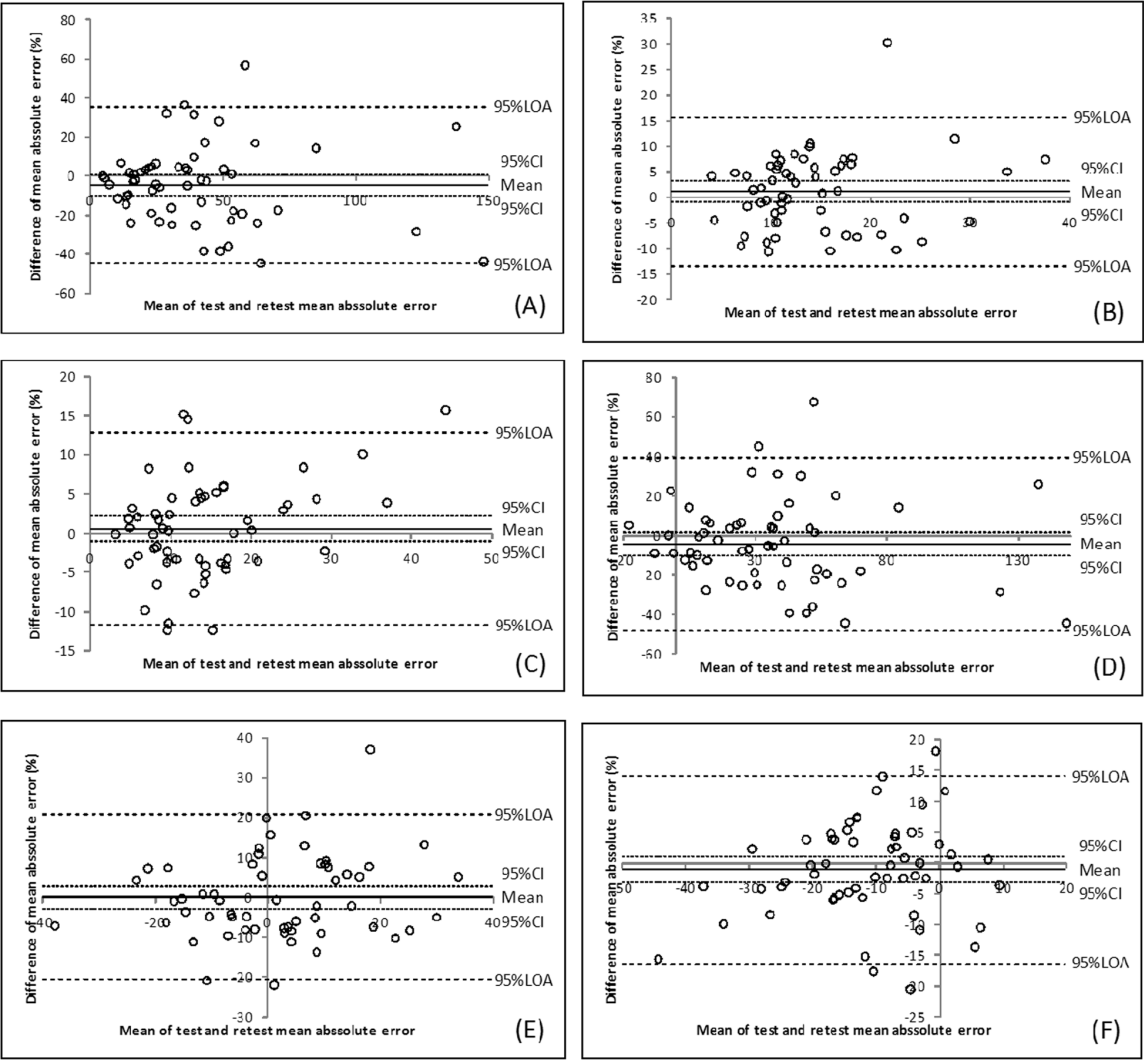
Bland–Altman plots for the AE at 10% MVIC (**a**), 30% MVIC (**b**), and 50% MVIC (**c**) and for the CE at 10% MVIC (**d**), 30% MVIC (**e**), and 50% MVIC (**f**) of the key pinch

**Fig. 5:**
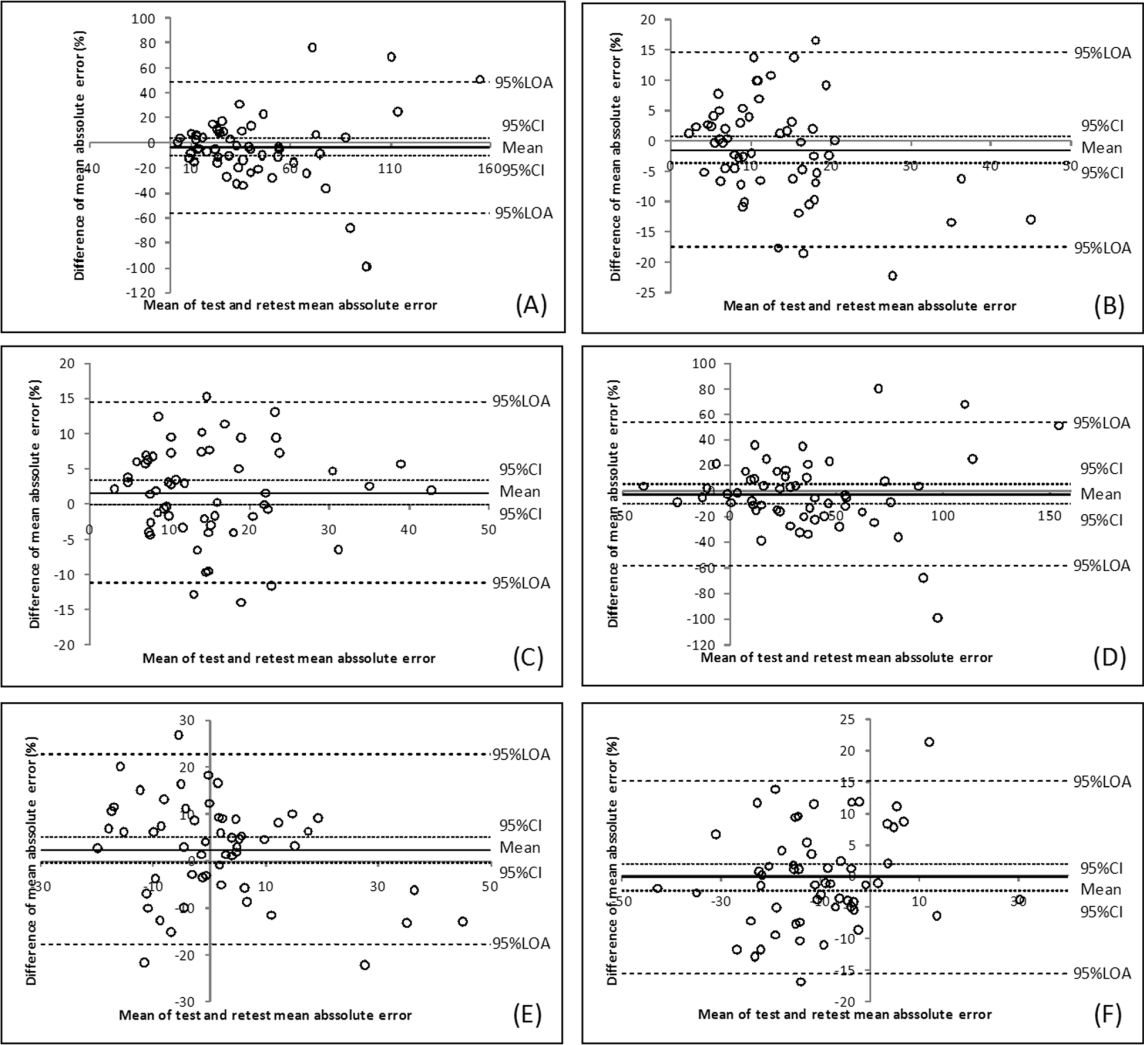
Bland–Altman plots for the AE at 10% MVIC (**a**), 30% MVIC (**b**), and 50% MVIC (**c**) and for the CE at 10% MVIC (**d**), 30% MVIC (**e**), and 50% MVIC (**f**) of the palmar pinch

The Bland–Altman plots are presented in Figs. 3, 4 and 5. The 30 and 50% MVIC results showed a narrow 95% LOA.

## Discussion

### Test–retest reliability

Previous studies have determined that the ICC of the hand grip force sense is moderately reliable (ICC = 0.704) [18]. Benjaminse claimed that good intersession reliability (ICC = 0.764) of the force sense in the hip joint was observed during flexion. He also claimed that the force sense in the hip in other planes were not reliable [16]. In the shoulders, the force sense measurement is very reliable (internal rotation: ICC = 0.981, external rotation: ICC = 0.978) [17]. Based on a literature survey, it was determined that the reliability of the pinch force sense has not been investigated. The acquired results indicated that the test-retest reliability of the pinch force sense in healthy subjects can generate stable and similar measurements at three force levels (10, 30, and 50% MVIC). Therefore, in this study, moderate reliability of the pinch force sense was determined to be acceptable.

Based on the high value of the ICC results in our study, the tip pinch and palmar pinch force sense test demonstrated good reliability, and the key pinch indicate fair to good reliability. To assess the measurement error magnitude, absolute reliability or agreement was considered [51]. SEM facilitates the quantification of absolute reliability and can be reported in the actual units of measurement. The SEMs at 30 and 50% MVIC were lower than that at 10% MVIC. The Bland–Altman plot analysis shows that the 95% CI includes the value zero. This result indicates that there was no statistically significant difference between test and retest. One value (1.8%) were outside the range of the 95% LOA range, as shown in Fig. 3 (E) and Fig. 4b. Two values (3.6%) were outside the range of the 95% LOA range, as shown in Fig. 4a, d and Fig. 5f. Three (5.4%) were outside the range of the 95% LOA, as shown in Fig. 3f, Fig. 4e, f, and Fig. 5e. Four (7.1%) were outside the range of the 95% LOA, as shown in Fig. 3a, b, c, d and Fig. 5b, c, d. Five (8.9%) were outside the range of the 95% LOA, as shown in Fig. 4c, and Fig. 5a. The mean difference was near zero, which indicates fair reproducibility.

The ranges of the 95% LOA for the 30 and 50% MVIC variables were narrower than those of the 10% MVIC variables. The SEM values and Bland–Altman plot revealed that the agreement and reproducibility of the 30 and 50% MVIC conditions were superior to those observed at 10% MVIC.

### Factors affecting reliability

Reliability can be affected by the experimental conditions and other variables that vary between the initial test and the retest. These variables include the effects of fatigue, learning, and memory on performance. To minimize the potential for any fatigue effects due to difficulties in concentrating, the subjects were allowed to rest for 30 s at the end of each trial and for 2 to 3 min after 5 trials [27]. The small improvement in the AE and CE in the second measurement may be indicative of an overall learning effect. The force reproduction task was practiced repeatedly until subjects reported that they were comfortable performing these estimates and that they understood all the instructions. This practice helped the participants understand the testing procedure and helped reduce the risk of a learning effect. Familiarization processes also have the potential to improve test reproducibility [52–55]. The order of the target forces was also randomized to avoid any learning effects associated with the limited test-retest time difference. To minimize the influence of learning or fatigue on the reproducibility of the study, the test and retest reliability sessions were separated by a period of 7 days. It has been confirmed in previous studies that longer intervals of 1–7 days between sessions can improve test-retest reliability [16, 28, 56]. The measurement of proprioception via pinch force sense ipsilateral testing is reliant upon the ability of the subjects to replicate the target force levels from memory [57, 58]. To minimize the potential for any memory effects due to difficulties in concentrating, the study was performed in a quiet room.

### Limitations

In this study, standardization of the pinch force sense procedure reduced the potential for differences in the conditions for the test and retest measurements. A high reliability was observed for the pinch force sense measurements in healthy adults. However, our study has some limitations. For example, the small sample size is insufficient to represent the population. In addition, only healthy young adults with a mean age of 18.6 years were enrolled in this study. Therefore, the excellent test-retest reliability in the study may only be valid for the assessment of pinch force sense in similarly aged and healthy individuals. Thus, additional studies are needed to examine these relationships among other age groups.

## Conclusion

The results confirm the fair to good reliability of the force reproduction test for measuring pinch force sense in healthy adults. The lower SEM values and the narrower 95% LOA in the AE and CE variables obtained for 30 and 50% MVIC compared with 10% MVIC indicate that they are more reliable. Based on the satisfactory reliability findings, we draw the following conclusions: 1) this study demonstrates that high test-retest reliability can be achieved using the standardized positioning and approach that is proposed. 2) According to the reliability of the measurements, 30 and 50% MVIC are the most reliable pinch force sense levels. Therefore, we recommend that the AE and CE variables at 30 and 50% MVIC are used in future studies of subjects without pathologies. When these recommendations are followed, this test appears to be a reliable means of assessing pinch force sense in healthy controls.

## Data Availability

Raw material can be provided upon request, please contact LL or YXL.

## Abbreviations

AE: Absolute error
CE: Constant error
CI: Confidence interval
CNS: Central nervous system
CTS: Carpal tunnel syndrome
ICC: Intraclass correlation coefficient
LOA: Limits of agreement
MSDs: Musculoskeletal disorders
MVIC: Maximal voluntary isometric contraction
SEM: Standard error of the measurement
